# Noise and Health

**Published:** 1920-12-11

**Authors:** 


### Noise and Health.

The Chelsea Saw Case which recently came before the Court attracted a good deal of public attention —partly because of the amusing nature of some of the evidence, and partly because the residents who applied for an injunction against the owners of the saw to put an end to the nuisance included an English judge. The decision in favour of the claimants seems to have aroused adverse comment among a certain class of people who appear to imagine that, because the objecting residents were in a large measure the well-to-do class, their complaints were the frivolous complaints of a leisured class who cared nothing for the important fact that this great electric saw was providing employment for a number of workmen On the contrary, however, the evidence showed that the complaint was well-justified, and one can imagine nothing more diabolically distracting on nerves not more than normally sensitive than the extraordinary noise produced by this saw, beginning suddenly at any time of the day, piercing the ears, shrieking, whining, moaning, and doing all the terrible things so vividly described by nerve-wracked witnesses.

It is perfectly true, as one commentator in the newspaper has remarked, that modern civilisation cannot be run without a great deal of noise; but it is equally true that the noise of modern civilisation in large populous centres is not in itself beneficial to the community, but distinctly harmful. There is a very real relation between noise and health. Modern "nerves" are, in part at least, the product of noise in modern civilisation. Ask any London doctor whether the shrieks and jars of the trains in tubes, the myriad noises of the streets, the sudden clangs and hoots of trams and motor-cars, and all the other compelling sounds of a great city are conducive either to long life or to that fine mental poise which gives zest as well as length to life. It is in all probability the case that many people who have never heard the Chelsea saw in full operation, and who have been laughing contemptuously at the "fuss" made by the local residents, are themselves disadvantageously affected by perpetual noises of which they are outwardly unconscious, but which makes them out of humour in the mornings, distract their general attitude towards life, and therefore diminish their efficiency as citizens. The noise of present-day existence reinforced by its atmosphere of bustle and countless little worries, has played a very considerable part in bringing about what is commonly described as the characteristic unrest of this age. It was not because they were unreasonable, but because they were intelligent that the Chelsea residents combined to resist the torture of the Chelsea saw.

